# Characteristic and Mechanistic Investigation of Stress-Assisted Microbiologically Influenced Corrosion of X80 Steel in Near-Neutral Solutions

**DOI:** 10.3390/ma16010390

**Published:** 2022-12-31

**Authors:** Huihua Guo, Rui Zhong, Bo Liu, Jike Yang, Zhiyong Liu, Cuiwei Du, Xiaogang Li

**Affiliations:** 1 Institute for Advanced Materials and Technology, University of Science and Technology Beijing, Beijing 100083, China; 2 Collaborative Innovation Center of Steel Technology, University of Science and Technology Beijing, Beijing 100083, China; 3 National Materials Corrosion and Protection Data Center, Beijing 100083, China; 4 Beijing Advanced Innovation Center for Materials Genome Engineering, University of Science and Technology Beijing, Beijing 100083, China

**Keywords:** SRB, MIC, X80, FIB

## Abstract

The behavior and mechanisms of the stress-assisted microbiologically influenced corrosion (MIC) of X80 pipeline steel induced by sulfate-reducing bacteria (SRB) were investigated using focused ion beam-scanning electron microscopy (FIB). Electrochemical results show that SRB and stress have a synergistic effect on the corrosion of X80 steel. SRB accelerated the transformation of Fe_3_O_4_ into iron-sulfur compounds and may have caused the film breakage of X80 steel products. The obtained FIB results provide direct evidence that SRB promotes the corrosion of X80 steel.

## 1. Introduction

Corrosion reduces the service life and represents a significant threat to the operational safety of oil and gas pipelines and other important infrastructure elements. Accidents related to corrosion perforation and cracking have caused substantial economic losses and many casualties. Microbiologically influenced corrosion (MIC) has been recognized as a serious threat to various metal structures. MIC accounts for 20% of corrosion damage in metal and building materials and represents an increasingly important branch of steel corrosion research [1,2,3].

Sulfate-reducing bacteria (SRB) play an important role in MIC [4,5], which is known to induce or accelerate pipeline steel corrosion. The MIC caused by SRB is often widely distributed and has the worst effects [6]. Since the 1960s, numerous studies on microbial corrosion caused by SRB have been carried out [7,8]. Alabbas et al. [9] compared the corrosion behavior of X80 pipeline steel irrespective of the presence of SRB. Their results showed that the corrosion rate of X80 pipeline steel in the presence of SRB was six times that observed in sterile conditions and that SRB greatly promoted the corrosion of pipeline steel. Many studies have since been carried out the investigating the mechanism anaerobic corrosion caused of the SRB [3,10,11,12,13], Von Wohlzogen Kuhr [14] proposed the famous cathode depolarization theory in 1934. Booth et al. [15] investigated the mechanism of microbial corrosion and elucidated a typical SRB anaerobic corrosion theory. Widely applied mechanisms include that related to cathodic depolarization, as well as concentration differential cell theory, anode fixation theory, and direct electron transfer theory [16,17].

In recent years, field cases and laboratory studies have shown [18,19,20] that SRB in the soil environment has an important impact on the stress corrosion cracking (SCC) behavior of X80 steel. Many tests at accident sites have shown that SRB was present under the peeled coating of failed pipelines [21,22]; this finding indicated that the synergistic effect of stress and SRB could accelerate the corrosion of X80 pipeline steel [23,24,25]. In April 2004, an API 5L X52 pipeline ruptured in northern Iran, resulting in an oil spill [18]. SRB was involved in and altered the pitting and crack origin of the pipeline steel, which was the main cause of the failure of the pipeline. Kim et al. [26] hypothesized that microorganisms could be used as microbial indicators to evaluate the extent of stress corrosion cracking in pipeline steel. Work by Wu et al. [27,28,29] showed that stress and SRB had a strong synergistic effect on promoting the corrosion of X80 steel. Xie et al. [30] compared the Tafel slope and the SRB growth curves and found that anodic reactions are fundamental to the stress corrosion cracking of X80 pipeline steel in the presence of SRB. SRB can also promote the anodic dissolution of X80 steel. Yang et al. [31] performed a mechanistic study on the stress-assisted MIC of 2205 duplex steel caused by SRB in a simulated seawater environment. Their results suggested that SRB might significantly influence the pitting behavior of 2205 DSS and that stress promotes the MIC behavior. Wei et al. [32] found that the stressed coupons underwent more severe SRB corrosion and pitting at the same locations under the simulated disbanded coating due to the mechanochemical effect. These results indicated that the pitting process was affected by the applied elastic stress in the X80 steel. So far, there have been a lot of research results on the corrosion of X80 steel under stress or SRB alone, but there is still a lack of reasonable explanation of the corrosion mechanism under the coupling of stress and SRB.

The majority of the current research on the synergistic effect of SRB and stress on corrosion remains focused on their effect on the properties of a given material and the associated macroscopic behavior rules. In these works, the synergistic effect of SRB and stress on the underlying corrosion mechanism has rarely been investigated. The focused ion beam-scanning electron microscope (FIB-SEM) is a powerful tool for studying microsystems. This tool allows for the continuous slicing of metals and reveals their cross-section and inner structure [33,34,35,36].

In this work, electrochemical and surface analysis methods were used to study the MIC mechanism of X80 steel caused by SRB under stress, and the influence of stress and SRB on X80 steel corrosion was determined. We could characterize the complete corrosion product layer and sample surface under the biofilm using FIB-SEM images without disrupting the structure of cells or the corrosion product layer. The bacteria distribution in the biofilms was obtained, and a comprehensive mechanism diagram was drawn that describes to describes how SRB and stress influence the corrosion of X80 steel.

## 2. Materials and Methods

### 2.1. Bacterium and Culture Medium

The SRB strain (*Desulfovibrio desulfuricans* ATCC 7757) was obtained from Northeastern University in China. The obtained bacterial solution was added to 50 mL of culture medium (ATCC 1249 liquid medium) at a ratio of 5 vol.%, as shown in Table 1 [37,38]. The pH of all three components was adjusted to 7.5 ± 0.2 via the addition of hydrochloric acid and sodium hydroxide. Component I was sterilized using a steam pressure cooker at 121 °C for 20 min and then cooled to 40 °C in the placement solution; components II and III were then added to component I in the aseptic table. Component IV was subsequently added to component I via filtration. Oxygen was then removed from the medium via the addition of N_2_ for a period of 4 h to prepare the sterilization medium. All containers and glass rods were sterilized using a steam pressure cooker at 121 °C for 20 min. All operations were carried out on an aseptic operating table. The bacteria transfer operation was carried out in an anaerobic chamber. The strain was cultured at 37 °C for 72 h and then stored in a refrigerator at −20 °C for use as bacterial seeds. The pH was measured using a 5 mL syringe to extract approximately 3 mL of liquid from the flask; this sample was then placed in a small centrifugal tube. The SRB quantity was calculated statistically using the hemocyte counter method, and its growth curve was established. The sterile medium for the corrosion tests was obtained by replacing the deionized water in the SRB culture medium with a nearly neutral simulated soil solution (NS4) [39]. A mixture of 95 vol.% N_2_ + 5 vol.% CO_2_ was then passed through the solution to remove the trace amounts of oxygen and prepare a neutral experimental environment. The SRB strain seed was inoculated into a sterile medium at a volume ratio of 5 vol.% in an anaerobic chamber to prepare a culture [34].

### 2.2. Sample Preparation

**Figure 1:**
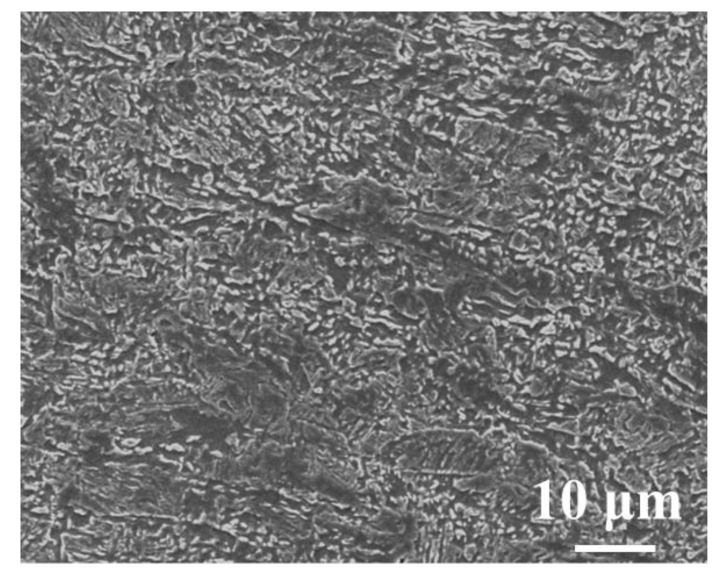
Metallographic diagram of X80 steel.

The chemical composition and the chemical properties of the X80 steel selected for this study are given in Table 2 and Table 3. The composition was measured by the Optical Emission Spectrometer (Hitachi Analytical Instruments Co. LTD, Shanghai, China) three times, and the average value was calculated. The metallographic diagram of X80 steel is shown in Figure 1. The typical granular bainite structure of X80 is mainly composed of ferrite and discontinuous carbon-rich martensite-austenite islands.

**Figure 2:**
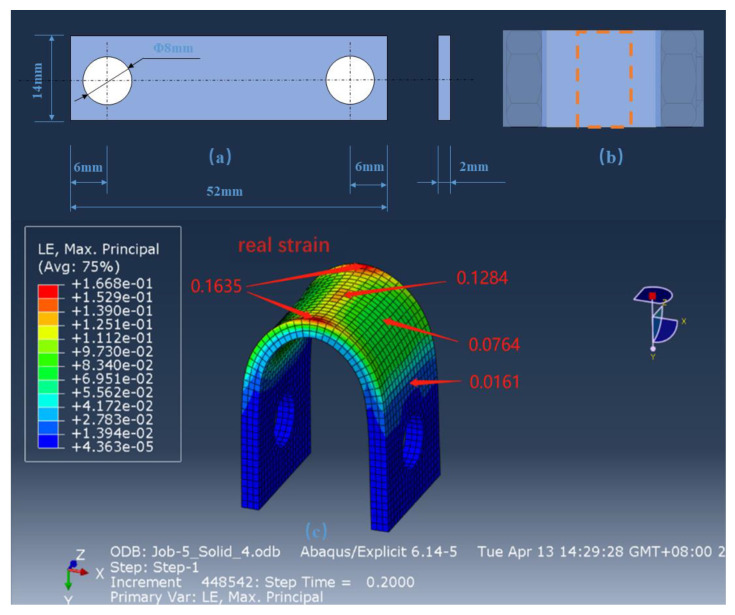
Processing dimension diagram of the U-bent sample of X80 steel (**a**) dimension diagram, (**b**) schematic diagram, and (**c**) strain distribution of the U-bent sample of X80 steel.

U-bend samples were prepared according to the ASTMG 30-2003 standard. A diagram showing the assembly and dimensions of the U-bent samples used here is shown in Figure 2a. All parts were machined from the same X80 steel. The observation surface and both sides of the flake sample were polished using sandpaper (2000 grit).

The sample was then bent by using a U-shaped sample prototype, and the opening angle of the sample was tightened and adjusted to 180° by stainless steel bolts to make the U-bent sample. The sample was separated from the bolts using polytetrafluoroethylene washers. The U-bend was fixed, the wire was soldered to the bolt, and the other areas were sealed with silica gel except for the working area of the arc top.

The electron backscatter diffraction (EBSD) was used to characterize the strain, and the EBSD sample was cut from the top of the U-bent sample to compare the strain of the top part of the U-bent samples quantitatively, as depicted in Figure 2b. The strain distribution in the U-bent sample was numerically analyzed using ABAQUS finite element analysis software, as shown in Figure 2c. As can be seen from Figure 1c, the maximum strain observed was 0.1635 near the edge of the U-shaped curve, a strain of 0.1284 was observed in the center of the arc top; a minimum strain of 0.0161 was observed in the region that exhibits minimal deformation, which is only one-tenth of the strain observed in the top edge of the arc.

**Figure 3:**
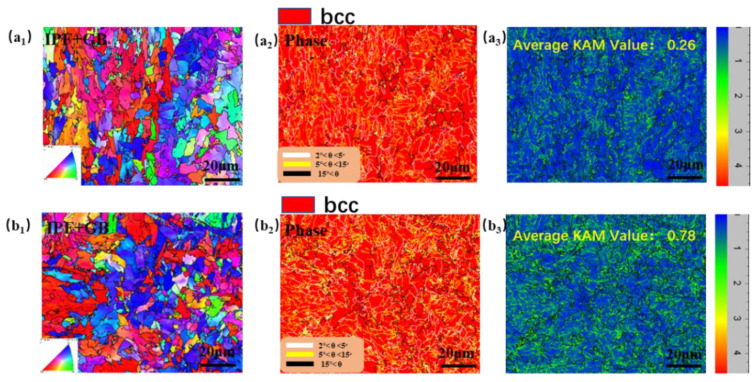
(**a_1_**)–(**a_3_**) are the Inverse Polar Figure map (IPFZ), the phase map, and the KAM map of the stress-free square sample, respectively. (**b_1_**)–(**b_3_**) are the Inverse Polar Figure map (IPFZ), the phase map, and the KAM map of the stressed sample, respectively.

The microstructure of X80 steel was characterized via EBSD (Figure 3). The EBSD samples were prepared by electrolytic polishing. The electrolytic polishing liquid was 85% alcohol + 10% perchloric acid + 5% glycerol. The average kernel misorientation (KAM) [40] is often used to characterize the degree of plastic deformation observed in a sample. As can be seen in Figure 3, the average KAM value at the top of the U-bent arc was 0.78, nearly three times the average KAM value in the absence of the applied stress of 0.26.

### 2.3. Immersion Tests

For the immersion experiment, X80 steel was cut into samples with dimensions of 10 × 10 × 2 mm^3^, and each surface was polished with increasingly fine sandpapers (800 girts). After ultrasonic cleaning, the steel was immersed in isopropyl alcohol for 2 h and exposed to ultraviolet irradiation for 1 h to sterilize the samples. The same batch of the medium was used in the same batch of experiments as far as possible. After inoculation, the medium solution was cultured at 30 °C for 14 days. In the experiment, the sterile medium was used to create a control group for the immersion experiment. All other experimental conditions were consistent between the two groups. Three parallel samples were set for each experiment group.

### 2.4. Surface Characterization

After removing the samples and stress-samples from the sterile medium and bacteria medium, they were immersed in 2.5% (V/V) glutaraldehyde solution for 8 h to fix a biofilm to the sample. Then, they were dehydrated in 50, 70, 80, 90%, 95 and 100% (V/V) ethanol solution [34]. Before the SEM observation, the sample surface was sprayed with gold to ensure good electrical conductivity. The surface corrosion morphology of the samples before and after removing any rust was observed via SEM (FEI Quanta 250, Thermo Fisher Technologies, Beijing, China), which was 20.00 kv of acceleration voltage. The composition of the surface corrosion products without stress was analyzed via X-ray photoelectron spectroscopy (XPS). The surface morphology of the samples was observed again after rust removal.

The bacteria were cultured in anaerobic condition at 37 °C for three days to reach a sufficiently high concentration. The bacteria were inoculated into 100 mL culture flasks at a ratio of 50% (V/V), and they were then placed in a shaker to promote their growth. 24 h later, the X80 U-bent test and X80 stress-free samples were put into culture bottles in an anaerobic chamber, and the culture bottle was put into an incubator at 37 °C for about 24 h. The samples were then taken out of the culture bottle, fixed, dehydrated, and air-dried for later use. When the U-bent samples were cut, the biofilm should not be removed. U-bent samples of X80 steel were soaked for 14 days before testing. FIB (Helios G4 UC plasma system) images were used to characterize the surface morphology distribution.

### 2.5. Electrochemical Test

The electrochemical curves were obtained using the Reference 600 Plus system. An anaerobic custom-made three-electrode system consisting of an X80 steel coupon as the working electrode, a platinum sheet as the auxiliary electrode, and a saturated calomel electrode (SCE) as a reference electrode was used. The cell dimensions were 500 mL with a broth volume of 200 mL and a headspace of 300 mL. First, the open circuit potential (OCP) was tested for 30 min to ensure the stability of the test system. A linear polarization resistance test was then carried out within the potential range of −10 to +10 mV vs. the OCP at a sweep rate of 0.125 mV/s. Gamry Echem Analyst software (Version 7.04) was used to calculate tangents near the OCP curves and thus obtain the polarization resistance (R_p_). The electrochemical impedance spectroscopy (EIS) tests were performed from 100 kHz to 0.01 Hz with a 10 mV AC signal amplitude. The ZSimpWin 3.50 software was used to fit EIS results with the proper equivalent circuits. Finally, the dynamic polarization curves were obtained at a scanning rate of 0.5 mV/s after 14-day immersion; the scanning range was from −1.3 to −0.4 V vs. SCE. The corrosion current density values (i*_corr_*) were obtained using the Tafel region extrapolation method.

## 3. Results

### 3.1. SRB Growth Characteristics

**Figure 4:**
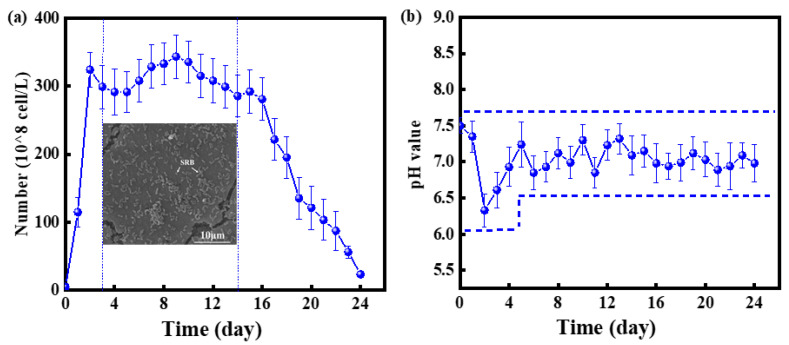
(**a**) The SRB growth curve of SRB in the culture medium (**b**) pH variation of SRB in the culture medium.

Figure 4 shows the growth curve and pH variation of SRB in the culture. SRB is elongated elliptical bacteria which are distributed on the surface of the steel matrix. The growth curve of SRB exhibits the typical three stages of bacterial growth: The first stage was a logarithmic growth period of 1–3 days; during this period, the bacteria rapidly proliferated in two-split mode; this period continued while the culture medium had surface space and energy for the bacterial growth.

In the second stage, which occurred from 4 to 14 days, stable growth occurred; the number of bacteria that could be contained in the culture medium reached its maximum, and the rate of bacterial growth and death were roughly the same. Finally, the third stage was observed in which the nutrients in the culture medium decreased, and the life cycle of the bacteria ended. The number of bacteria decreased rapidly in this period. The pH changes of the SRB-containing medium during this experiment are shown in Figure 4. The pH of the solution decreased slightly during the first two days, which may be caused by organic acid, the metabolite of SRB. The solution pH remained unchanged after 2 days, which may be related to the buffer pairs in the bacterial metabolites. These results indicate that SRB did not reduce the pH of the matrix solution and promoted the corrosion of the steel matrix in this manner.

### 3.2. Observation and Analysis of Corrosion Products

**Figure 5:**
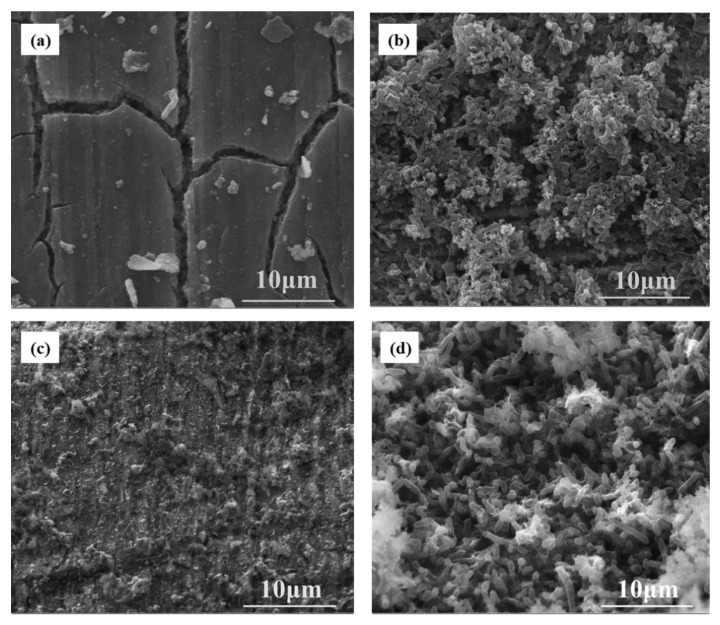
SEM image of surface corrosion products of X80 steel sample immersed for 14 days (**a**) sterile and stress-free (**b**) bacteria and stress-free (**c**) sterile and stress (**d**) bacteria and stress.

The corrosion products found on the surface of the X80 steel matrix after soaking for 14 days are shown in Figure 5. Figure 5a shows the morphology of corrosion products on the surface of X80 steel under aseptic and stress-free conditions. In this case, a cracked but relatively complete product film can be seen. The cracks may be caused by dehydration. Figure 5b shows the surface morphology of X80 steel that was exposed to bacteria but free of stress. Sheets of biofilm covered the steel matrix, and SRB was distributed in clusters and mixed with extracellular secretions. Figure 5c shows the morphology of the corrosion products on the surface of X80 steel in aseptic conditions but subject to stress. It can be seen that some corrosion precipitates exist on the surface. Figure 5d shows the morphology of the corrosion products on the surface of the X80 steel that was subject to both bacteria and stress. It can be seen that SRBs are abundant and densely distributed on the substrate product film; the density of the SRB was higher than that observed on the steel subject to bacteria in the absence of stress.

**Figure 6:**
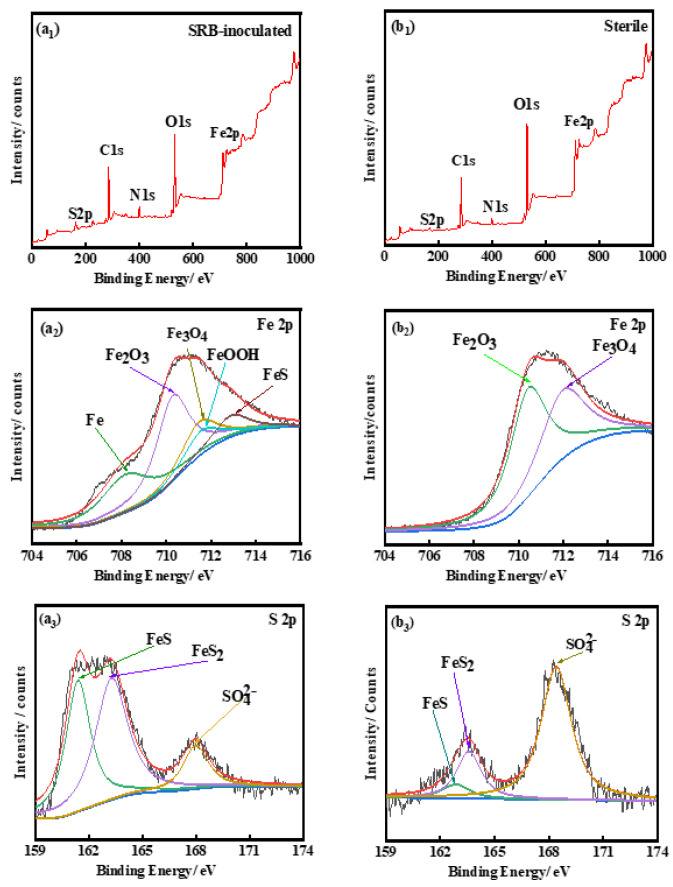
XPS survey spectra (**a_1_**,**b_1_**), detailed spectra of Fe (**a_2_**,**b_2_**), S (**a_3_**,**b_3_**) in SRB culture medium (it starts with a) and in the sterile medium (it starts with b).

**Figure 7:**
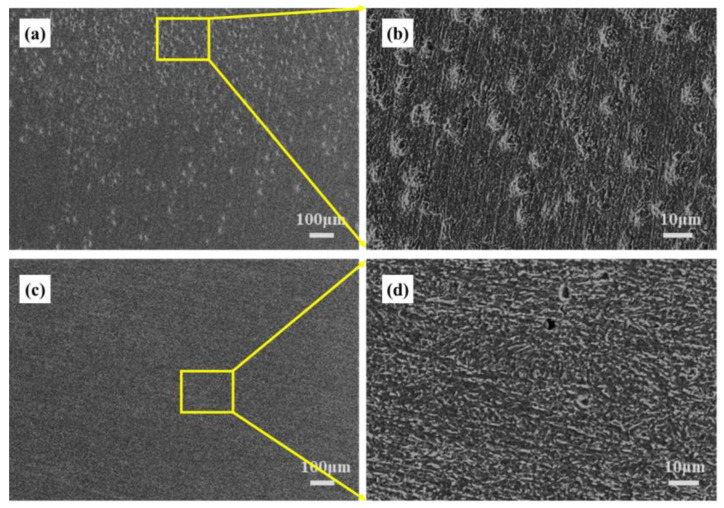
SEM image of X80 steel sample immersed for 14 days after derusting surface (**a**) bacteria and stress-free (**b**) partial enlargement of Figure (**a**); (**c**) sterile and stress-free (**d**) partial enlargement of Figure (**c**).

XPS was used to characterize the corrosion products on the surface of the test specimens. The test results are shown in Figure 6. In Figure 6a_1_–a_3_, the full spectrum of XPS, the partial peaks of Fe, and the partial peaks of S are shown, respectively, for the samples subjected to bacteria. In Figure 6b_1_–b_3_, the full XPS spectrum, the partial peaks of Fe, and the partial peaks of S are shown, respectively, for the samples subjected to sterile conditions. As shown in Figure 6a_2_, the spectrum of Fe 2p of samples subject to the bacterial solution is characterized by five peaks at 708.7, 711.0, 711.4, 712.0, and 713.0 eV, corresponding to Fe, Fe_2_O_3_, Fe_3_O_4_, FeOOH, and FeS, respectively [41]. The presence of FeS on the substrate surface indicates that SRB participated in the corrosion process of X80 steel samples. After 14 days of immersion, the product film on the surface of the X80 steel substrate was found to be incomplete, and Fe could be directly detected in the XPS tests. However, in Figure 6b_2_, only Fe_2_O_3_ and Fe_3_O_4_ were detected in the Fe 2p spectrum for the samples soaked in the aseptic solution; Fe and FeS were not detected, indicating that the passivation film formed on the surface of the X80 steel in an aseptic environment was relatively complete and that the content proportion of sulfate anions was higher than that observed in the samples subject to bacteria. In Figure 6a_3_, it can be observed that the S 2p spectrum could be fitted with three peaks at 162.3, 163.6, and 168.4 eV, corresponding to FeS, FeS_2_, and SO_4_^2−^, respectively, and the content of FeS and FeS_2_ were much higher than that of hexavalent sulfur. FeS_2_ is not common in general iron and sulfur corrosion products, and the S-peak was not obvious in the samples subject to a sterile environment in Figure 6b_3_, although these three components were also detected. These results indicate that SRB was involved in the corrosion process of X80 steel. The product film formed on the surface of X80 was broken, and its corrosion resistance decreased. After the samples were soaked in sterile and bacteria-bearing medium solutions for 14 days, the corrosion products of the sample were then removed. The surface of the sample after rust removal is shown in Figure 7. In Figure 7a,b, the surface of X80 steel is subject to bacteria, but no stress is shown; dense corrosion pits and significant local corrosion can be observed in these figures. As can be seen from Figure 7c,d, which corresponds to the samples after 14 days of immersion, the surface of the X80 steel matrix in a sterile stress-free environment exhibited a small amount of pitting and no obvious local corrosion.

**Figure 8:**
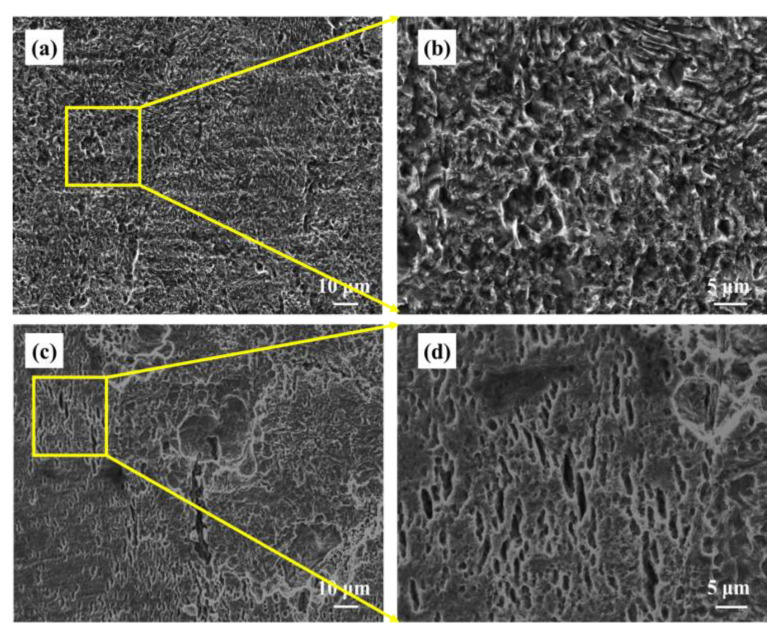
SEM image of X80 steel U-bent sample immersed for 14 days (**a**) sterile and stress; (**b**) partial enlargement of Figure (**a**); (**c**) bacterial and stress; (**d**) partial enlargement of Figure (**c**).

The X80 steel U-bent sample was soaked in sterile and bacteria-bearing medium solutions for 14 days. Then the corrosion products of the sample were then removed. The surfaces of the top part of the curve samples are shown in Figure 8. Figure 8a shows the surface of the X80 steel U-bent sample subject to bacteria. It can be seen that the surface corrosion was not significant and neither pitting, nor cracks were present in Figure 8a. In the corresponding enlarged view, shown in Figure 8b, it can be seen that the initiation of microcracks and stress corrosion was not particularly pronounced. Figure 8c shows the morphology of the X80 steel that has been subject to an environment with bacteria and subject to stress after rust removal; this figure shows patches of pitting pits and cracks covering the surface; a significant level of local and stress corrosion can be seen.

**Figure 9:**
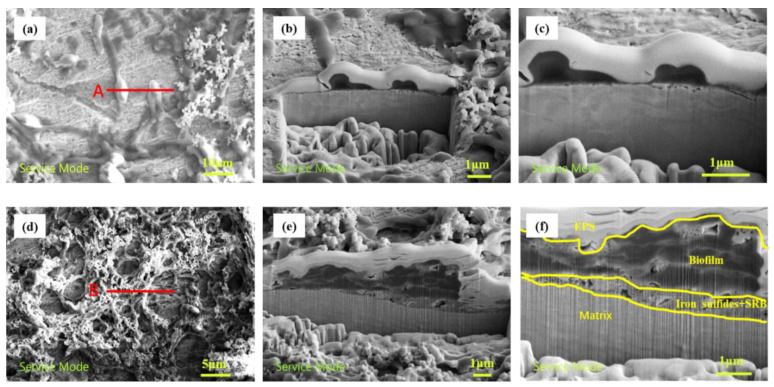
FIB section of stress-free sample; (**a**) top view of single bacterium; (**b**) FIB cross-section of single bacteria; (**c**) partial enlargement of Figure (**b**); (**d**) top view of the colony; (**e**) FIB section of the colony; (**f**) biofilm distribution of colony.

After the samples that were not subject to stress had been immersed for 24 h, FIB-SEM was used to characterize the samples, as shown in Figure 9. In Figure 9a, every single bacterium was rod-shaped, and the cross-section was cut in the direction indicated by the red line labeled A. In Figure 9c, it can be observed that there are some very tiny gaps and black spots between the matrix and SRB; these features may generate a loose iron sulfide layer and lead to the initiation of pitting. However, there were no black spots on the matrix surface of the samples without SRB, indicating that SRB promoted the pitting of X80 steel. A piece of biofilm can be seen in Figure 9d. The cross-section was cut in the position indicated by the red line labeled B. It can be seen that the biofilm could be roughly divided into three layers, as shown in Figure 9f: The top layer was made up of white bacteria extracellular secretion. In the middle, a dense and thick film layer formed by bacteria, metabolites, and corrosion products can be observed; this layer provides a degree of protection for the matrix. The bottom layer of the corrosion product layer was porous. Li [10] studied the MIC pitting mechanism via FIB-SEM and found that C and O were found in the upper layer of the biofilm, which are the main component of proteins and other organic matter. The results showed that the upper layer was mainly extracellular secretions and the corrosion product of the Fe and S observed on the substrate surface was iron sulfide. The C, O, S, and P elements were the primary elements in bacterial cells, indicating that there were more cells in the pits. It can be inferred that the primary component of the loose and porous corrosion layer was iron sulfide.

**Figure 10:**
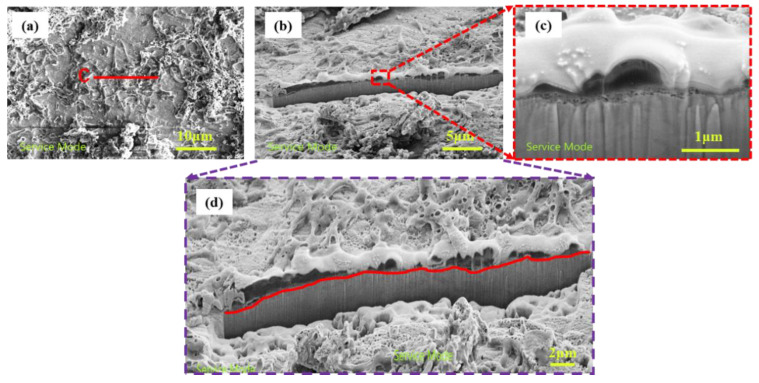
FIB section of a bacterial thin region of stress sample; (**a**) top view of the bacterial thin area; (**b**) FIB cross-section of the bacterial thin region; (**c**) enlarged cross-section of bacteria thin area; (**d**) enlarged biofilm-matrix interface.

Figure 10 shows the FIB characterization of the sample subjected to stress after immersion for 24 h. This characterization sample was made up of the thin biofilm area from the U-bent of the sample shown in Figure 10a transected along the red line labeled C; it can be seen that in this area, the surface was surrounded by a white secretion. There was a porous membrane layer below the bacteria shown in Figure 10c, and the membrane layer below the surface of the substrate was not very neat due to the dissolution of the surface iron, indicating pitting corrosion.

**Figure 11:**
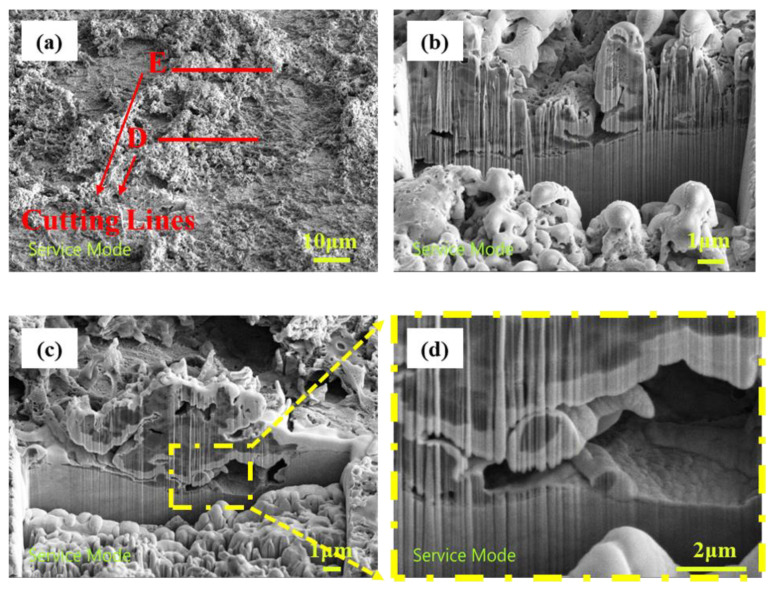
FIB section of a thick bacterial region of stress sample; (**a**) top view of bacteria thick area; (**b**) section D of the thick region of bacteria; (**c**) section E of thick bacterial region; (**d**) enlarged view of section E of the thick region of bacteria.

A thick biofilm area of the U-bent sample in Figure 11 was cut along the red lines labeled D and E. In this area, a thick biological membrane and a biofilm surface clearance hole could be observed covering the substrate surface in Figure 11b. A larger pore was observed between the biological membrane and the substrate in Figure 11c, and the pore was a relatively closed space: in this figure, the upper region shows a continuous membrane layer, and the lower region shows the substrate; a large number of bacteria were present in this region. It is hypothesized that SRB penetrated the base of the biofilm, promoting the dissolution of iron.

The above observations indicate that after 24 h soaking in the culture medium, SRB survived on the matrix surface of the matrix and promoted the pitting initiation on the sample that was not subjected to stress. Meanwhile, SRB may continue to move in the gap on the surface of the matrix and promote the dissolution of the X80 steel matrix. The surface product layer in the stressed sample was damaged. The existence of the strain may make the substrate dissolve more readily and promote the products of the surface layer to become discontinuous. The bacteria activity was more intense, which may further break the integrity of the product layer and accelerated the rate of the cell gaining electrons.

### 3.3. Electrochemical Results

**Figure 12:**
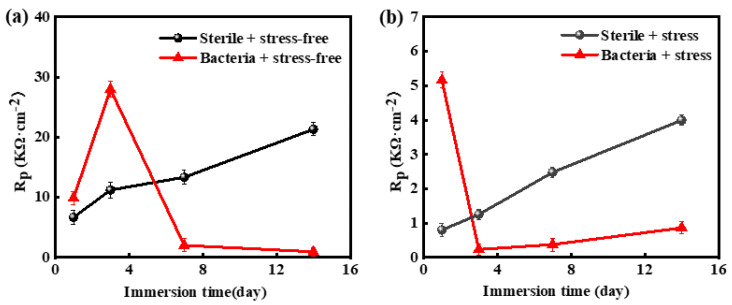
Change of Rp value of time: (**a**) X80 square sample stress-free sample; (**b**) X80 U bending stress sample.

Figure 12 shows the variation in the polarization resistance (R_p_) values of the X80 steel samples that were not subject to and subject to stress in SRB and SRB-free media for a given soaking period. In the case of the X80 steel sample in the sterile medium, the value of Rp continued to increase, and the corrosion resistance increased correspondingly. It indicates that the corrosion rate of X80 steel in a sterile environment decreases continuously, due to the corrosion products deposited on the surface of the sample protecting the matrix.

The Rp value of the X80 steel sample in the SRB medium increased over the initial period of 1–3 days, which was due to the more corrosion products that were generated to protect the matrix. In this case, SRB propagation promotes the formation of a thicker layer of corrosion products and inhibits corrosion. In the 3–7 days period, the value of Rp began to decrease quickly. On the one hand, the proliferation of bacteria destroyed the integrity of the corrosion product layer. On the other hand, the SRB can “eat electrons” to absorb energy directly to accelerate corrosion. After the seventh day, the curve drops at a lower rate. Although the concentration of SRB reached the highest in the 7–14 days, the inhibition effect of the corrosion product layer and the promotion effect of SRB activity keep a relative balance. Of course, the corrosion promotion effect of SRB biofilm is greater. Therefore, overall, SRB promotes the corrosion process of the X80 steel. It can be seen that the value of Rp for the X80 steel sample subject to bacteria is significantly lower than that of the sample exposed to sterile conditions after soaking for 14 days.

In the aseptic environment, the value of R_p_ of the stressed X80 steel sample showed a steady increase, but its value of R_p_ was always smaller than that of the sample in the aseptic stress-free condition. The stressed specimen subject to the bacteria showed a high R_p_ value in the first two days because the protective film was complete at first. On the third day of the immersion test, the value of R_p_ decreased to a minimum and maintained a relatively low level as the bacteria multiplied. Under the joint action of microorganisms and stress, thick and dense product film was formed on the substrate surface in less than one day, so the Rp value measured on the first day was the largest. With the active SRB, the integrity of the product film was rapidly destroyed, SRB rapidly multiplied, and its Rp value reached the minimum on the third day. After that, the Rp value increased slowly, which was still far less than that under the aseptic stress condition.

**Figure 13:**
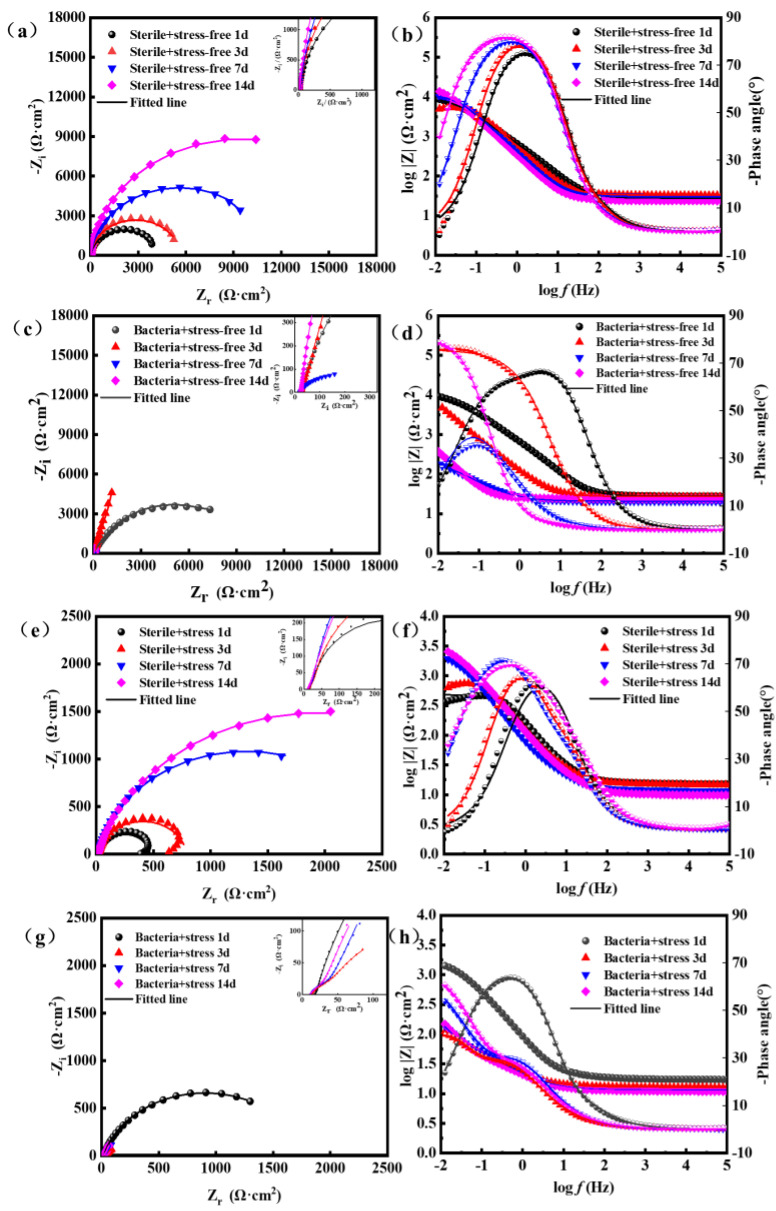
Nyquist and Bode plot values of the X-80 coupons immersed in the different culture media for different durations: (**a**,**b**) Sterile + Stress−free; (**c**,**d**) Bacteria + Stress−free; (**e**,**f**) Sterile + Stress; (**g**,**h**) Bacteria + Stress.

**Figure 14:**
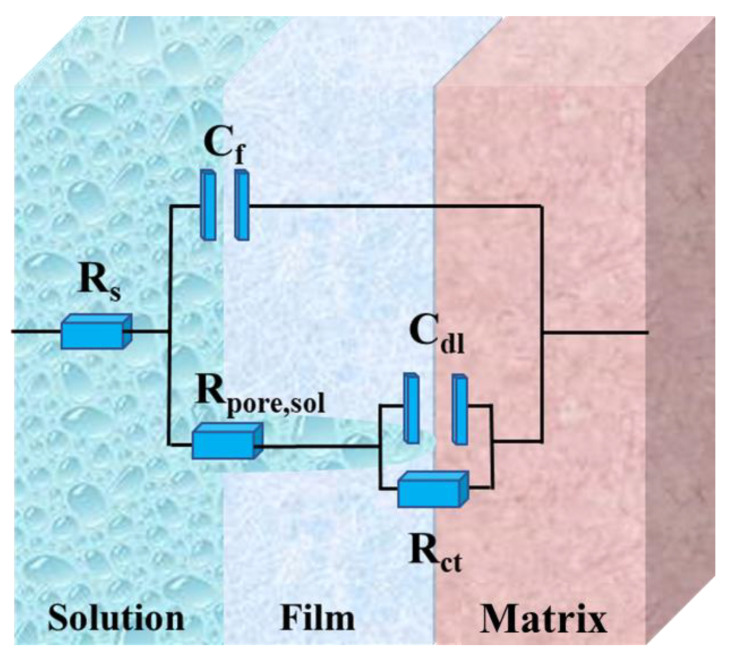
Equivalent circuits for EIS fitting.

The electrochemical impedance spectrum test results of the X80 steel sample are shown in Figure 13. The radius of the Nyquist plot in the immersion experiment of sterile medium solution (a,b) kept increasing. According to the Nyquist diagram of the immersion experiment in medium bacterial solution (c,d), the arc radius of the low-frequency area increased in the first 3 days, and then decreased in the third to seventh days. In the corresponding Bode figure, the impedance modulus value of the low-frequency region of the X80 steel sample in the sterile medium continued to maintain an upward trend, while the overall trend was downward in the culture medium with bacteria. The corresponding phase Angle curve has only a high-frequency time constant, which represents the product layer on the sample surface. The time constants in the low-frequency region represent the charge transfer process in the metal and solution interface double layer. The fitted value and equivalent circuits are shown in Table 4 and Figure 14. In Figure 14, R_s_ represents the solution resistance; C_f_ represents the constant phase Angle element (CPE) of the corrosion product and biofilm mixture layer, R_pore.sol_ represents the main ion current solution resistance of the pore channel, and C_dl_ and R_ct_ represent the double-layer CPE and charge transfer resistance at the metal matrix and solution interface, respectively [42].

**Figure 15:**
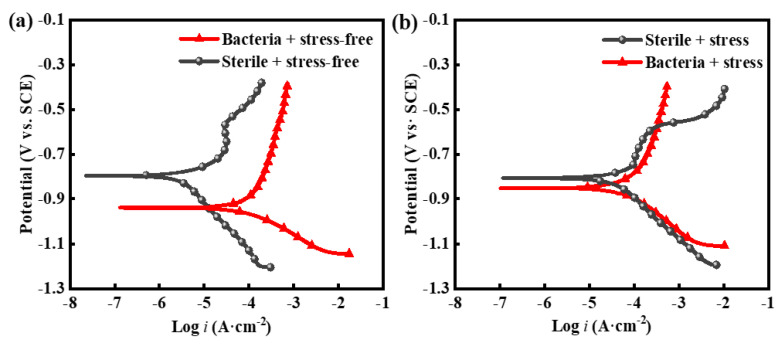
Potentiodynamic polarization curve of X80 steel sample soaked for 14 days: (**a**) X80 square sample stress-free sample; (**b**) X80 U bending stress sample.

Figure 15 shows the potentiodynamic polarization curves of the X80 steel stress-free and stressed samples soaked in the sterile and bacterial-bearing medium solution for 14 days; these values were obtained via a Tafel fitting shown in Table 5. The potentiodynamic polarization curves of the U-bent samples with bacteria showed a significantly increased corrosion current density of 124 μA/cm^2^ (i_1_). This value was much higher than that of the corrosion current density of the sterile U-bent sample, which took a value of 52.6 μA/cm^2^ (i_2_). The corrosion current density of the sample subject to bacteria but no stress after soaking for 14 days was 122 μA/cm^2^ (i_3_), indicating that the stress had little effect on the corrosion of the X80 steel when the sample was subject to both stress and SRB for 14 days. In the sterile stress-free system, the corrosion current after soaking for 14 days was only 4.67 μA/cm^2^ (i_4_). Thus, in the sterile condition, the stress had a significant influence on the corrosion of the X80 steel samples; in this case, the corrosion current was more than ten times greater. In summary, it was found that i_1_ > i_3_ > i_2_ > i_4_. The effect of SRB alone on the corrosion of X80 steel is clear, and the effect of stress alone on the corrosion of the X80 steel is also significant, but the effect of the stress is not as strong as that of SRB.

## 4. Discussion

### 4.1. Influence and the MIC Mechanism of SRB on Corrosion of X80 Steel

According to the biocatalytic enhancement sulfate reduction (BCSR) theory proposed by Gu [1], the corrosion process linked with SRB can be divided into two types. The first type, extracellular electron transfer (EET), refers to the transfer of electrons from the metal surface to the bacterial cytoplasm either directly or via an electron carrier, known as direct electron transfer and mediated electron transfer, respectively. The second type, metabolite MIC, refers to corrosion that is caused by secreted metabolites. The anodic process is the process of iron dissolution, oxidation, and electron loss:

Fe → Fe^2+^ + 2e^−^ (1)

It is hypothesized that the bacteria use the electrons released by iron oxidation. The terminal electron acceptor is the sulfate inside the bacteria:

SO_4_^2−^ + 9 H^+^ + 8 e^−^ → HS^−^ + 4 H_2_O (2)

Electrons are transferred from the metal surface to the c-cytochrome protein bound by the outer membrane such that SRB can use these electrons for sulfate reduction and energy generation [43,44]. The BCSR theory has been supported by many subsequent experiments under the condition of carbon starvation [43,45,46]. Figure 3b shows that the minimum pH of the SRB inoculated medium was approximately 6.0, and the pH remained close to 7.0; thus, it can be inferred that the local pH was insufficient to cause corrosion. Fe_2_O_3_ and Fe_3_O_4_ were detected in the Fe2p spectrum via XPS, the samples subject to the aseptic conditions, but Fe and FeS were not detected in these samples, indicating that the passivation film formed on the surface of X80 steel in a sterile environment was relatively complete and dense. The FeS and FeS_2_ content was higher in the bacteria group. The physiological activities of SRB degraded the integrity of the protective layer on the surface of the matrix. Gu [2] found that SRB induces MIC in this case by using Fe^0^ as an electron donor. Bacteria can directly “eat electrons” to absorb energy and thus cause considerable pitting corrosion on the matrix [1]. The FIB-SEM results also provided direct evidence that there were some very small gaps and spots under the bacteria, see Figure 9e, which may be made up of a loose iron sulfide layer, which leads to pitting initiation. The sample surfaces without SRB do not exhibit these black spots.

### 4.2. Synergistic Effect Mechanism between Stress and SRB on X80 Steel

After the U-bent sample had been immersed for 14 days, it was found that many cracks had formed on the X80 steel that was subject to the bacteria (see Figure 7d), whereas only a few microcracks were seen in the sterile group (see Figure 8b). The FIB-SEM results also directly indicated that the presence of SRB greatly increases the pitting sensitivity of the U-bent sample. The extent of iron dissolution, pitting, and cracking on the surface was largely dependent on the presence of SRB, and the loose product layer on the matrix surface of the stressed sample was severely damaged. On the one hand, the matrix was more soluble because of the applied strain, and the product layer on the surface was not complete, which led to an increase in the activities of bacteria. In this way, the SRB metabolism is elevated, and the integrity of the product layer is further broken. The electrochemical results show that SRB and stress have synergistic effects on the corrosion of X80 steel. Based on Gutman’s mechanical–chemical interaction theory and microbial energetics and corrosion electrochemistry theory, Wu [25,27,28] gave a thermodynamic and kinetic explanation of SRB cracking. In the presence of SRB, the corrosion driving force of ferric alloys increases, and it also increases under the action of stress. It is also calculated that the stress and SRB physiological activity have a synergistic effect in accelerating the initiation and propagation of cracks in Ferro-metallic materials.

The existence of SRB on the substrate surface can promote the initiation of pitting; the bacteria can live in the loose space on the substrate surface and promote the dissolution of the X80 steel matrix. Stress is found to accelerate the rate of MIC; more “nutrients” are supplied to SRB for propagation. The pitting corrosion of X80 steel caused by SRB leads to stress concentration. The formation of FeS on the surface of the pipeline steel was detected on the samples subject to bacteria. Sulfides can promote the mechanical–electrochemical interaction on the pipeline steel and thus accelerate the initiation of pitting on the surface of the pipeline steel [30]. At the same time, the physiological activity of SRB produced S^2−^, HS^−^ and H_2_S, which promoted hydrogen penetration.

According to the mechanical-electrochemical theory [47], the presence of stress increases the electrochemical thermodynamic activity of steel. The stress affects the microstructure of X80 steel, and the change in grain size and grain boundary orientation angle will affect the electrochemical activity of X80 steel [32]. Stress promotes dislocation movement, diffusion, and blockage, which promote the occurrence and spread of pitting. Cracks are initiated and spread at the pitting corrosion site.

**Figure 16:**
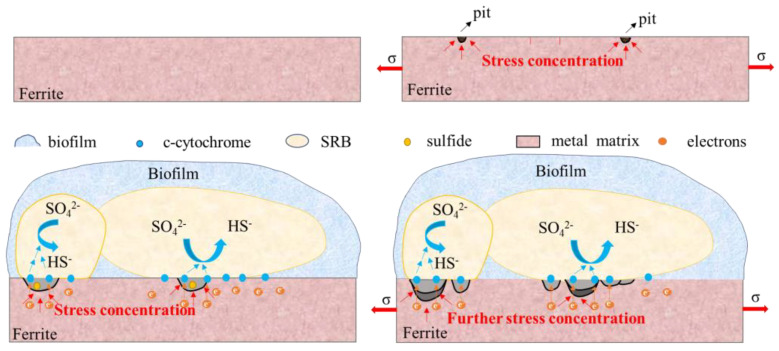
A comprehensive mechanism diagram to describe the interaction of SRB and stress on X80 steel corrosion.

The synergy between SRB and stress was found to Increase the sensitivity of corrosion. SRB on the surface of the substrate can simultaneously carry out direct EET, promoting the dissolution of iron and thus accelerating the corrosion of pipeline steel. The combined action of stress and SRB results in increased corrosion and pitting rates of pipeline steel. The mechanism behind this phenomenon is shown in Figure 16.

## 5. Conclusions

This study investigated the stress-assisted MIC mechanism of X80 steel in an SRB-inoculated medium via various electrochemical and surface analysis methods. The primary conclusions of this work are as follows:

(1) The obtained FIB-SEM results provide direct evidence that SRB promotes the corrosion of X80 steel. The physiological activity of SRB produces sulfides and destroys the integrity of the protective layer on the substrate surface.
(2) The physiological activity of SRB, as well as sulfides and stress, can increase the corrosion in X80 steel. The corrosion rate of samples at different conditions follows the order as Exposed to both bacteria and stress > Subjected to bacteria but without stress > Subjected to stress in a sterile condition > Not subject to stress or bacteria.
(3) We proposed a comprehensive mechanism that describes the interaction between SRB and stress: SRB and stress together accelerate the corrosion of pipeline steel. SRB on the substrate surface can simultaneously perform direct EET and promote iron dissolution. Stress affects the microstructure of X80, leading to stress concentration.

## Figures and Tables

**Table 1:** Components of ATCC 1249 medium.

	Chemicals	Amount
Component I	MgSO_4_·7 H_2_O	2.0 g
	NH_4_Cl	1.0 g
	Na_3_C_6_H_5_O_7_ (trisodium citrate)	5.0 g
	CaSO_4_	1.0 g
	Distilled Water	400 mL
Component II	K_2_HPO_4_	0.5 g
	Distilled Water	200 mL
Component III	NaC_3_H_5_O_3_ (sodium lactate)	3.5 g
	Yeast Extract	1.0 g
	Distilled Water	400 mL
Component IV	Filter-sterilized 5% (wt.) (NH_4_)_2_ Fe (SO_4_)_2_ (ferrous ammonium sulfate). Add 0.1 mL of this solution to 5.0 mL of medium prior to inoculation.	

**Table 2:** Composition of X80 steel.

Element	C	Mn	Ni	Cu	Nb	Fe
Amount (wt.%)	0.0717	1.78	0.17	0.121	0.084	/

**Table 3:** Chemical properties of X80 steel.

	Yield Strength	Tensile Strength	Elongation	Vickers Hardness
X80	743 MPa	885 MPa	13%	265.47 HV

**Table 4:** Fitting parameters of the EIS spectra of the unloaded and loaded coupons in sterile medium and SRB culture medium after different exposure periods.

	R_s_ (Ω·cm^2^)	Y_a_ (Ω^−1^·cm^−2^·s^n^)	n _a_	R_pore,sol_ (Ω·cm^−2^)	C_dl_ (Ω^−1^·cm^−2^·s^n^)	n _dl_	R_ct_ (Ω·cm^−2^)	Chsq
Sterile + stress-free								
1 day	33.39	1.650 × 10^−4^	0.9616	2.403 × 10^1^	1.261 × 10^−4^	0.9454	4.060 × 10^3^	8.11 × 10^−4^
3 days	32.97	1.796 × 10^−4^	0.9815	33.12 × 10^1^	1.205 × 10^−4^	0.9572	5.585 × 10^3^	1.25 × 10^−3^
7 days	30.14	2.921 × 10^−4^	0.9480	23.57 × 10^1^	1.200 × 10^−4^	0.9702	1.096 × 10^4^	6.36 × 10^−4^
14 days	24.06	2.643 × 10^−4^	1.0000	11.32 × 10^1^	2.521 × 10^−4^	0.8997	1.923 × 10^4^	1.01 × 10^−4^
Bacteria + stress-free								
1 day	28.01	2.4830 × 10^−4^	0.8839	1679 × 10^3^	2.078 × 10^−4^	0.7669	8.208 × 10^3^	1.84 × 10^−4^
3 days	27.16	1.8740 × 10^−4^	0.8395	1656 × 10^3^	2.757 × 10^−3^	0.8234	3.406 × 10^4^	4.33 × 10^−4^
7 days	20.82	2.2400 × 10^−6^	0.7551	11.50 × 10^2^	2.385 × 10^−3^	0.9582	1.016 × 10^2^	1.25 × 10^−3^
14 days	23.65	1.3700 × 10^−6^	0.9079	2.854 × 10^3^	2.298 × 10^−3^	0.9656	8.532 × 10^3^	2.80 × 10^−3^
Sterile + stress								
1 day	15.50	6.959 × 10^−3^	0.9355	61.98 × 10^3^	4.025 × 10^−4^	0.9875	4.001 × 10^2^	2.82 × 10^−3^
3 days	14.22	1.126 × 10^−3^	0.9016	62.67 × 10^3^	7.849 × 10^−4^	0.9818	7.943 × 10^2^	1.58 × 10^−3^
7 days	11.75	1.889 × 10^−3^	0.8469	68.25 × 10^3^	8.001 × 10^−4^	0.9948	2.561 × 10^3^	1.58 × 10^−4^
14 days	9.988	1.613 × 10^−3^	0.8108	103.7 × 10^3^	2.669 × 10^−4^	0.9844	3.606 × 10^3^	1.66 × 10^−4^
Bacteria + stress								
1 day	26.7	1.582 × 10^−4^	0.4582	1188 × 10^3^	7.253 × 10^−6^	1.0000	3.915 × 10^3^	2.07 × 10^−3^
3 days	12.56	3.378 × 10^−2^	0.5212	0.018 × 10^3^	1.402 × 10^−11^	0.7156	8.026 × 10^2^	1.41 × 10^−3^
7 days	11.32	1.768 × 10^−2^	0.6972	56.36 × 10^3^	4.483 × 10^−2^	0.8585	1.207 × 10^3^	3.60 × 10^−5^
14 days	10.91	2.358 × 10^−2^	0.6503	61.32 × 10^3^	2.574 × 10^−2^	0.9019	1.202 × 10^16^	7.30 × 10^−5^

**Table 5:** Comparison table of Tafel fitting results of 14-day polarization curve of X80 steel.

	Sterile + Stress-Free	Bacteria + Stress-Free	Sterile + Stress	Bacteria + Stress
i*_corr_* (μA·cm^−2^)	4.67	122	52.6	124
E*_corr_* (mV_vs SCE_)	−797	−939	−807	−852

## Data Availability

The authors declare that no new data were created.
